# Childhood adverse life events and parental psychopathology as risk factors for bipolar disorder

**DOI:** 10.1038/tp.2016.201

**Published:** 2016-10-25

**Authors:** V Bergink, J T Larsen, M H J Hillegers, S K Dahl, H Stevens, P B Mortensen, L Petersen, T Munk-Olsen

**Affiliations:** 1 The National Centre for Register-Based Research, Department of Economics and Business Economics, Aarhus University, Aarhus, Denmark; 2 Erasmus Medical Centre, Department of Psychiatry, Rotterdam, The Netherlands; 3 Lundbeck Foundation Initiative for Integrative Psychiatric Research, iPSYCH, Aarhus University, Aarhus, Denmark; 4 University Medical Center Utrecht, Brain Center Rudolf Magnus, Utrecht, The Netherlands; 5 Department Of Sociology and Social Work, Aalborg University, Aalborg, Denmark; 6 North Jutland Police, Aalborg, Denmark; 7 Centre for Integrated Register-Based Research, Aarhus University, Aarhus, Denmark

## Abstract

Childhood adverse events are risk factors for later bipolar disorder. We quantified the risks for a later diagnosis of bipolar disorder after exposure to adverse life events in children with and without parental psychopathology. This register-based population cohort study included all persons born in Denmark from 1980 to 1998 (980 554 persons). Adversities before age 15 years were: familial disruption; parental somatic illness; any parental psychopathology; parental labour market exclusion; parental imprisonment; placement in out-of-home care; and parental natural and unnatural death. We calculated risk estimates of each of these eight life events as single exposure and risk estimates for exposure to multiple life events. Main outcome variable was a diagnosis of bipolar disorder after the age of 15 years, analysed with Cox proportional hazard regression. Single exposure to most of the investigated adversities were associated with increased risk for bipolar disorder, exceptions were parental somatic illness and parental natural death. By far the strongest risk factor for bipolar disorder in our study was any mental disorder in the parent (hazard ratio 3.53; 95% confidence interval 2.73–4.53) and the additional effects of life events on bipolar risk were limited. An effect of early adverse life events on bipolar risk later in life was mainly observed in children without parental psychopathology. Our findings do not exclude early-life events as possible risk factors, but challenge the concept of adversities as important independent determinants of bipolar disorder in genetically vulnerable individuals.

## Introduction

Bipolar disorder is a complex, severe and multifactorial mood disorder.^1^ The estimated heritability of bipolar disorder is high and the evidence of genetic risk factors for the first onset of the disease is convincing.^2, 3^ However, in clinical practice, only a minority of patients report a positive family history for bipolar disorder. Instead, the occurrence of any psychopathology in the family is common, which is in agreement with the overlapping genetic vulnerability between bipolar disorder and other disorders (for example, unipolar depression, anxiety disorders, substance abuse, attention-deficit hyperactivity disorder, non-affective psychotic disorders).^4^ In addition, patients with bipolar disorder frequently report on early-life events and the impact of these events on the onset and course of their disease. This is of great importance since epigenetic studies suggest that early-life events potentiate genetic vulnerability and act as important determinants for the first clinical manifestation of the disease.^5^

Importantly, the majority of life events cannot be regarded as independent from genetic vulnerability for mood disorders, in particular parental psychopathology can be considered an early-life stressor as well as a marker of a genetic liability to mental health problems. The susceptibility to bipolar disorder is most likely due to an interaction between genetic and environmental risk factors (including early-life events), which challenges the design of studies on both early-life events and parental psychopathology.

Thus far, life events that are most investigated include severe life events such as childhood trauma and loss of a parent. Studies report that childhood trauma is associated with an increased risk for the first episode of bipolar disorder, including clinical characteristics like an earlier onset, rapid cycling course, higher rates of suicidality and more psychotic features.^6, 7, 8, 9, 10, 11, 12, 13, 14, 15, 16, 17, 18^ For the most part, prior studies have used semi-structured interviews or self-report measures to elicit information on early adverse life events. One concern for such study designs is the potential for selection- and reporting bias and the extent to which answers are affected by the current mood state of the responder.^19^ Population-based studies overcome this bias and have confirmed the increase in risk of bipolar disorder after early parental loss^20, 21, 22^ and childhood maltreatment.^23^ To date, very few studies have investigated the impact of less severe, but very common early-life events (for example, family disruption) on bipolar onset in the offspring.

Accordingly, the primary aim of the current study was to investigate the occurrence of a wide range of adverse life events during childhood varying in both frequency and severity, and the risk of bipolar disorder. More specifically, we included both highly prevalent life events (familial disruption, parental psychiatric and somatic illness and parental labour market exclusion) as well as less prevalent but severe life events (parental imprisonment, parental loss and placement in foster care). We used information from nation-wide registers for which prospectively and independently collected exposure data were available, and this large population-based cohort enabled us to:

1) Study associations between various adverse life events before age 15 years and subsequent risk of bipolar disorder by estimating the independent contribution of each single life event, as well as the risk increase after exposure to multiple life events. 2) Study to which extent family history of mental disorders increases risk of bipolar disorder and if childhood adverse life events add to this risk.

## Materials and methods

### Study population and design

To study the proposed aims we designed an epidemiological population-based cohort study including all persons born in Denmark from 1 January 1980 to 31 December 1998 to parents who were also born in Denmark. In summary, we defined the main exposure variables as a panel of various early-life events in children from 0 to 15 years, and the outcome of interest was defined as a registered diagnosis of bipolar disorder.

A total of 980 554 persons were included in the study cohort, after exclusion of people emigrating or dying or diagnosed with bipolar disorder before the age of 15 years. The follow-up time started at individual cohort members' 15th birthday, and follow-up ended at date of emigration, date of death, date of first recorded bipolar affective diagnoses or 31 December 2013, whichever came first. This provided a maximum follow-up period of 19 years and a maximum age of 34 years for the people in the cohort.

All citizens in Denmark have a personal identification number, which can be used to link information within and between registers, and this is essential for conducting register-based studies. For the present study, we linked each child to their legal parents through The Central Registration System, a register that holds updated information on vital status, migration and links to family members.

All records in the Danish health system are registered using ICD-8 codes until 1994, and ICD-10 codes from 1994 and onwards. For the present study, the included information came from a range of data sources/population registers. Note, as various population registers were initiated at different time points, we defined our study population by including children born 1980 or later, to ensure as complete exposure information as possible.

### Exposure: childhood adverse life events

The selected various childhood adverse life events we identified as our exposure variables were: (1) familial disruption, (2) parental somatic illness, (3) parental mental illness, (4) parental labour market exclusion, (5) parental imprisonment, placement in out-of-home care,^24^ and parental loss (natural or unnatural causes of death).^25^ Notably, we considered the inclusion of the adversity ‘sexual, physical or mental abuse', but there was not sufficient statistical power to conduct the analyses on this particular exposure.

Familial disruption was considered to be any other household composition than the cohortee living with both legal parents. Records were assessed through The Danish Civil Registration System^20^ where legal parenthood is not updated until as late as 1 year after childbirth and hence full records in immediate continuation of birth were not available. Familial disruption was considered an exposure if occurring at any time from proband's 1st to 15th birthday.

The Charlson Comorbidity Index (CCI) composed the underlying basis for assessing parental chronic somatic disorders.^26^ First registered admission as either in- or outpatient with any CCI diagnosis in The National Patient Register^21^ was regarded an exposure. We excluded all parental psychiatric diagnoses used in the CCI, as these were already encompassed in records of parental psychopathology.

Parental psychopathology was defined as parents' first registered admission as an in- or outpatient with any psychiatric diagnosis (ICD-8: 290-315; ICD-10: F00-F99) in The Psychiatric Central Research Register after birth of the child.^25^ Notably, primary care diagnoses are not captured and parents within the ‘no psychopathology' might have consulted their general practicioner with psychiatric symptoms. Importantly, every in- and outpatient contacts at psychiatric treatment facilities were detected and included.

Using data from the register on Integrated Database for Labour Market Research (IDA), we assessed parental labour market exclusion. Exposure was first record of the parent being outside the workforce after birth of the proband until age 15 years. Any of the following codes were included in the variable and indicate a permanent exclusion from the workforce; post-employment benefit (code 50), transition fee (code 55), pensioner (code 92), senior (code 92), early retirement pensioner (code 93) and official's pension (code 94).

Parental imprisonment was assessed through The National Crime Register held by Statistics Denmark. Unconditional sentences according to The Danish Penal Law, the Law on Psychedelic Drugs, the Offensive Weapons Act and the Law on Drink Driving was included as an exposure of adversity.

Records on placements of probands in out-of-home care were obtained through the register on Support for Marginalised Children and Adolescents.^24^ The following records formed the exposure of placement in foster care: family foster care; network foster family; familial placement; other foster family; municipal foster family; 24-h care centre; 24-h care centre with guarded ward, other 24-h care centre, acute 24-h care centre, partly closed 24-h care centre, social pediatric commune, boarding school, after school classes or continuation school, private room or the like, naval project, municipal 24 h initiative.

Records on parental loss were obtained through The Danish Civil Registration System.^27^ Parental loss was divided into natural or unnatural causes of death, based on information from the Register of Causes of Death.^28^ In cases of unknown cause of death of the parent, cohortees were excluded particularly for the sub analyses of the effects of parental loss.

### Outcome: bipolar disorder

The outcome of interest in this study was first occurrence of an in- or outpatient contact at a psychiatric treatment facility with a diagnosis of bipolar disorder (ICD-8: 296.09, 296.29, 298.09, 300.49. ICD-10: F30-F31). We identified and quantified the proportion of patients with bipolar diagnoses, who had preceding diagnoses of unipolar depression (ICD-8: 296.09, 296.29, 298.09, 300.49, ICD-10: F32, F33). Data on diagnoses were obtained through The Danish Psychiatric Central Research Register.^25^ All persons with bipolar disorder diagnoses before the age of 15 years were excluded from the study.

### Statistical analyses

We conducted survival analyses using Cox proportional hazard regressions, with the main outcome measure being hazard ratios (HRs). All estimates were adjusted for age in the nonparametric part of the model and for calendar time as a time-dependent variable categorized as 1995–1999, 2000–2004, 2005–2009 and 2010–2013, and for sex by means of separate underlying baseline hazards. Calendar periods were split into the aforementioned intervals in order to improve comparability of registrations over time. For all analyses, the reference group defined for comparison included children not exposed to any of the defined early-life events.

To account for correlation between the studied adversities we conducted separate analyses for children experiencing a single adversity compared with that particular adversity in combination with at least one of the other adversities. To present absolute risks of bipolar disorder, we calculated cumulative incidences for disease onset for each of the defined childhood life events by performing competing risk regression to take the competing event of death into account. Note, in order to protect the anonymity of individuals in our cohort study, the curves presented based on these analysis are smoothed to prevent identification of specific age at time of bipolar diagnosis.

To account for any possible modifying effect of history of parental psychopathology, we stratified analyses on two groups: children with and children without parents with mental disorders, respectively. For these stratified analyses, the variable ‘history of parental psychopathology' was defined as life-time parental psychopathology considering all diagnoses, also before the child was born. This variable was defined as a time-dependent covariate. Additional analyses where performed for children with one and both parents diagnosed with mental disorders.

Note that parental psychopathology was treated both as an early-life adversity (parental psychiatry as exposure defined as a first record after birth) and as a possible modifier (life-time history of parental psychopathology).

Additional sensitivity analyses included information on all cohort members with records of childhood and adolescence psychiatric disorders, which was obtained through The Psychiatric Central Research Register and exposure was measured given first contact as an in- or outpatient with any psychiatric diagnosis (except bipolar disorder) before age 15 years (ICD-8: 290-315; ICD-10: F00-F99). Further, we conducted additional sensitivity analyses, restricting our outcome of interest to mania only (all F30 codes except F30.0).

All analyses were conducted using STATA statistical software, version 13 (Stata, College Station, TX, USA).^25^

## Results

### Bipolar diagnosis

In total, 980 554 individuals were included in this study providing collectively representing 8 888 119 person-years. During follow-up 2235 cohortees received a diagnosis of bipolar disorder (1392 females and 843 males). For the 2235 cases in the cohort, the median age was 23 years (interquartile range 20–27). Please note that the statistics for age at diagnosis are mainly driven by the design of the study: all individuals were followed up from 15th birthday with a maximum follow-up of until 34 years of age, resulting in a relatively young cohort.

Of all cohortees with a bipolar diagnosis, 686 cases (30.7%) debuted with a registered diagnosis of depression at a median age of 21 years (interquartile range 18–25). They had a manic or hypomanic episode median 3 years after the initial depressive episode. Accordingly, in 1549 cohortees, (hypo) mania was the first officially registered episode of bipolar disorder.

### Adverse life events during childhood and the risk of bipolar disorder

Experiencing an early-life event was more commonly observed among patients diagnosed with bipolar disorder compared with the background population and among the bipolar cases it was more common to have experienced more than one life event (Table 1). We found a linear dose–response relation between number of adverse life events and HR for bipolar disorder as increasing number of early adverse events resulted in increasing risks of bipolar disorder (Table 2). Moreover, exposure to life events at a younger age resulted in increasing risks of bipolar disorder (Table 2).

Comparing each individual early adverse life event, no single event was associated to significantly differing HRs for bipolar disorder between females and males (data not shown). Of the 980 554 people (2235 cases) in the cohort, 35 592 (185 cases) had a psychiatric diagnosis other than bipolar disorder before age 15 years. These 35 592 people were included in the sensitivity analysis, but lack of statistical power impedes any conclusions about this subgroup. Moreover, 59 people had a diagnosis of bipolar disorder before the age of 15 years. We conducted additional sensitivity analyses, restricting our outcome of interest to mania only but did not have enough statistical power to detect any differences between the two outcomes: bipolar disorder broadly defined versus mania only.

**Figure 1:**
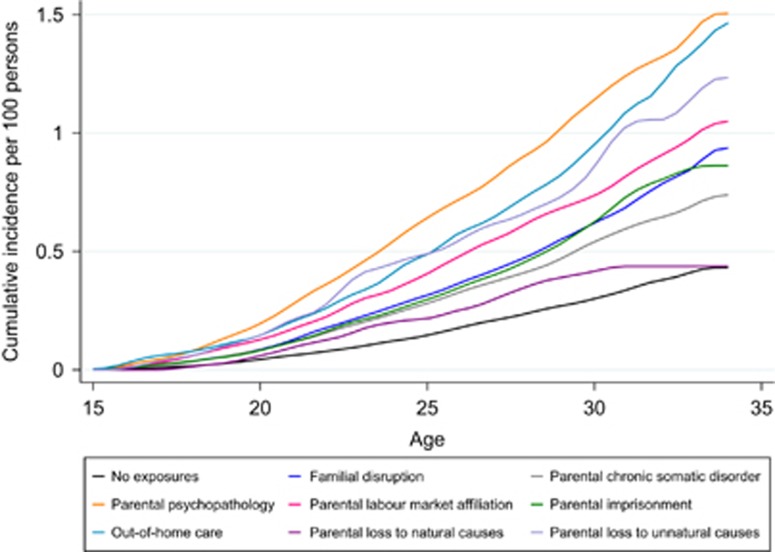
Cumulative Incidence for Bipolar Disorder after age 15 years over Number of Early Adverse Events. The cumulative incidence at age 34 years for each life event were: no exposure 0.43 95% CI 0.39–0.47; Familial disruption 0.94 95% CI 0.85–1.03; Parental chronic somatic disorder 0.74 95% CI 0.64–0.85; Parental psychopathology 1.51 95% CI 1.31–1.73; Parental labour market affiliation 1.05 95% CI 0.89–1.23; Parental imprisonment 0.86 95% CI 0.72–1.03; Out-of-home care 1.47 95% CI 1.17–1.82; Parental loss to natural causes 0.44 95% CI 0.31–0.60; Parental loss to unnatural causes 1.23 95% CI 0.81–1.82. CI, confidence interval.

Figure 1 shows the cumulative incidence of bipolar disorder after exposure to each of the eight individual life events. We could not identify specific ages with increased bipolar onset as the incidence was quite constant across follow-up. This finding might be driven by the maximum follow-up of cohortees until 34 years.

**Figure 2:**
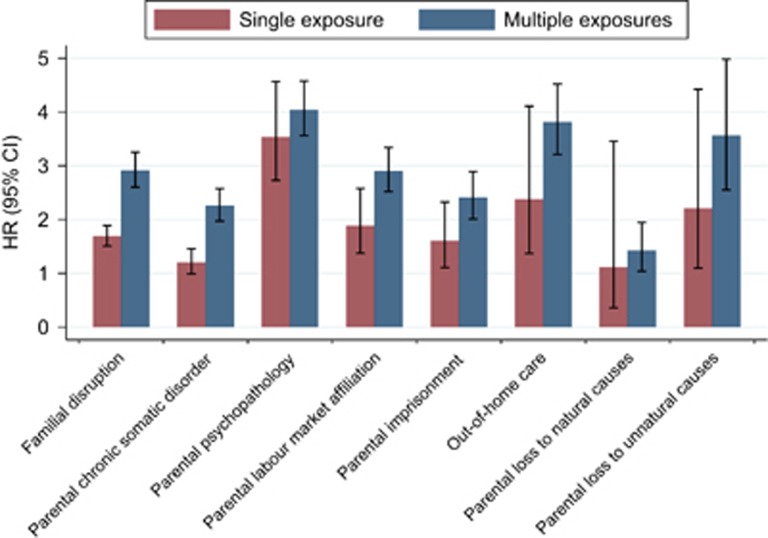
Bipolar risk after exposure to single and multiple early adverse life events. The blue bars show bipolar risk for each life event if it was combined with one or more other life events. The red bars indicate the independent risk of bipolar disorder for each of the eight life events. CI, confidence interval; HR, hazard ratio.

In Figure 2, we show risk estimates for a diagnosis of bipolar disorder later in life of each separate life event both as single exposure (red bars), and as multiple exposures in combination with at least one other type of exposure (blue bars). Specific results of the most prevalent observed early-life events: family disruption; parental somatic illness; and parental psychopathology are highlighted below.

### Family disruption

Family disruption was the most common life event, with a prevalence of 36% in the entire cohort. Family disruption was associated with increased risk of a later diagnosis of bipolar disorder, both as single exposure (HR 1.69; 95% CI 1.51–1.89) or multiple exposure (family disruption+one or more other life events, HR 2.91; 95% CI 2.60–3.25, Figure 2).

### Parental somatic illness

Somatic illness of one of the parents occurred in around 18% of cohortees. This life event as single exposure was not associated with an increased risk for bipolar disorder. In contrast, somatic illness was associated with increased risk for bipolar disorder if it was combined with one or more other life events (HR 2.25; 95% CI 1.97–2.58, Figure 2). The most severe outcome of parental somatic illness is natural death of one of the parents, which around 2% of cohortees experienced. Both single and multiple exposure to parental natural death was not associated with an increased risk of bipolar disorder later in life (Figure 2).

### Psychopathology in the parents

Psychopathology in the parents as single exposure greatly influenced the risk of a later diagnosis of bipolar disorder. If all environmental life events were absent and psychopathology in one or both parents was the only exposure before the age of 15 years, the HR was 3.53 (95% CI 2.73–4.53; Figure 2). More specifically, 136 007 cohortees (620 cases) had one parent with psychopathology and 13 817 (92 cases) had two parents with psychopathology. The hazard ratios for parental psychopathology as single exposure were 2.92 (95% CI 3.53–7.72) for one parent and 7.72 (95% CI 4.62–12.88) for two parents.

Table 2 shows the effects of a history of parental psychopathology and life events. If parental psychopathology is present, there is limited additional effect of exposure to life events (see also Supplementary Table 1: Hazard ratios for bipolar risk after exposure to early-life events. stratified by parental psychopathology status).

## Discussion

This population-based study gives an overview of the risk of bipolar disorder after the occurrence of one or more adverse life events before the age of 15 years. Half of the population was unexposed to any of these life events during childhood, which overall was protective for a diagnosis of bipolar disorder later in life. The most prevalent early-life events were family disruption, parental somatic illness and parental psychopathology and among these, having a parent with psychiatric illness was by far associated with the highest risk of bipolar disorder later in life. Of note, parental psychopathology included not only bipolar disorder, but any psychiatric diagnosis.

The observed increased risk of bipolar disorder in offspring of parents with psychopathology is not surprising, given that psychopathology in parents can be considered an early-life stressor as well as a marker of a genetic liability to mental health problems. Psychiatric symptoms in parents might impact family functioning and lead to a less cohesive environment, which makes offspring vulnerable to the development of psychopathology. Alternatively, being genetically predisposed to bipolar disorder might increase the risk of experiencing a traumatic event during childhood.^29^ The presence of childhood emotional or behavioral problems as early manifestation of bipolar disorder could increase family stress levels and lower the threshold for adverse life events. Interestingly, our findings suggest that parental psychopathology has a strong independent effect on the risk of bipolar disorder later in life. In those cases where all other seven environmental adverse events were absent, the risk in offspring for bipolar disorder was highly increased (HR 3.53; 95% CI 2.73–4.57). In children with parental psychopathology, additional effects of life events on bipolar risk were limited. Our findings add to evidence that there is a major role of genetic factors in bipolar disorder, especially in cases with an early onset. Adoption studies have shown that bipolar disorder is more common among the biological parents than the adoptive parents of bipolar adoptees^30^and twin studies show a markedly elevated concordance rate of bipolar disorder in monozygotic twins compared to dizygotic twins. Moreover, family studies have consistently found a high rate of bipolar disorder specifically among the relatives of early-onset cases.^31, 32^

However, based on the available data we cannot quantify any possible long-term negative effects of parental psychopathology leading to subtle chronic environmental stress for the offspring, as a result of dysfunctional parenting skills. Therefore, our study cannot fully disentangle whether our findings are the result of heritability, environmental influences or both.

In sharp contrast to parental psychiatric disease, somatic diseases in parents did not increase the risk for bipolar disorder later in life. Adding to this, there was no increased risk of bipolar disorder in cohort members who had experienced early parental loss, which is the most severe outcome of medical disease, and it is therefore unlikely that even severe parental somatic disease increases the risk.

Single exposure to the adversities ‘familial disruption, parental labour market exclusion, parental imprisonment and placement in foster care' were all associated with increased risk for bipolar disorder and the risk increased after combined exposure with other life events. Family disruption was the most common life event (HR 1.69; 95% CI 1.51–1.89). An increased risk after single exposure to family disruption is an important finding given the high prevalence of family disruption in the general population, and there are multiple possible explanations for this association. Theoretically, the altered composition of the family could have increased the risk. After divorce, children grew up either in a household with a single parent or in a household with one biological parent and a new partner, but our study was not designed to differentiate between these groups. Notably, after natural death of a parent, children encounter a similar situation; either they grow up in a single parent household or together with a stepparent. In these cases, there was no increase in bipolar risk later in life, which suggests that the increase in risk is driven by other factors, such as growing up in a conflictual stressful environment. An alternative explanation could be that parents are more likely to end their relationships if one of the two or both have subthreshold bipolar mood symptoms. Core features of subthreshold bipolar symptoms include mood swings, an overactive lifestyle and impulsive decision making alternated by periods of inactivity and passivity. These traits are not only associated with the occurrence of divorce or separation, but also with other life events such as losing a job or criminality.^33, 34^

Numerous previous studies have demonstrated an association between other stressful life events and the first onset as well as the recurrence of bipolar mood episodes.^21, 22, 35, 36, 37, 38^ A major issue when interpreting and comparing these studies, is the retrospective data collection, which complicates reliable reporting of both life events and mood episodes due to recall bias.^19, 39, 40^ This present study was designed to overcome this bias and confirmed in a population-based cohort an influence of various childhood adversities on the risk of a first bipolar mood episode. Other strength in our design are the separate analyses for children experiencing a single adversity compared with that particular adversity in combination with other adversities. Finally, we were able to stratify for parental psychopathology. Notably, effect sizes were relatively small for the effect of single adverse life events and the effect of adverse life events was very limited in children with parental psychopathology.

Thus far, childhood abuse is the most investigated adverse event.^18^ Studies report that childhood abuse is associated with an increased risk for the first episode of bipolar disorder, including clinical characteristics like an earlier onset, rapid cycling course, higher rates of suicidality and more psychotic features.^6, 11, 15, 16, 17, 23^ In our study, there was not sufficient statistical power to conduct the analyses on the adversity ‘sexual, physical or mental abuse', which is a limitation of our study. Other limitations of our study are the risk of misclassifications of both exposures (adversities) and outcomes (bipolar disorder). For example, there is a risk that the group of parents that have been classified as ‘no psychopathology' might still have been diagnosed with psychiatric symptoms in primary care but escaped detection. Similarly, children who have reported hypomanic symptoms to their general practitioner are not reported as cases in the registers covering contacts to psychiatric treatment facilities.

A few previous studies have investigated the role of life events specifically in offspring of parents with bipolar disorder. Overall, these studies showed an increased risk of bipolar spectrum disorders in offspring and they identified high numbers of life events in bipolar offspring.^14, 41, 42, 43, 44, 45, 46^ In comparison, our study was designed to look at overall effects of *any type* of parental mental disorder, because of the well-described genetic overlap between bipolar and other psychiatric disorders.^4, 47^ We found that broadly defined parental psychopathology is by far the most important risk factor for bipolar disorder in the offspring. The risk increase added to this by adverse life events during childhood was modest, albeit statistically significant. Our findings suggest that bipolar disorder is a disease with a strong genetic loading and challenge the concept of early-life events as important determinants particularly in genetic vulnerable individuals.

## Figures and Tables

**Table 1:** Numbers and percentages of cohort members exposed to early-life event exposures for the entire cohort and for bipolar cases

	*Cohort*		*Bipolar cases*	
	N*=980 554*		*N=2235*	
	*n*	*%*	*n*	*%*
*Exposures—age 0–14 years*				
Family disruption	350 987	35.8	1 068	47.8
Parental somatic illness	177 244	18.1	427	19.1
Parental psychopathology	84 665	8.6	434	19.4
Parental labour market exclusion	76 362	7.8	307	13.7
Parental criminality	45 023	4.6	168	7.5
Placement in out-of-home care	29 326	3.0	173	7.7
Parental natural death	19 859	2.0	44	2.0
Parental unnatural death	8385	0.9	44	2.0
				
*Any exposure*				
Age 0–4 years	259 377	26.5	844	37.8
Age 5–9 years	180 042	18.4	549	24.6
Age 10–14 years	184 711	18.8	538	24.1
Age 0–14 years	501 710	51.2	1 451	64.9

*Number of exposures—age 0–14 years*				
0	478 844	48.8	784	35.1
1	350 567	35.8	898	40.2
2	108 965	11.1	375	16.8
3	31 976	3.3	138	6.2
4+	10 202	1.0	40	1.8

Subanalyses included age-group specific effects of any adversity for children aged 0–4, 5–9, 10–14 and 0–14 years. To evaluate the effect of an increasing number of different adversities on bipolar risk. cohort members were grouped according to number of adversities experienced and subanalysis subsequently described the effect of experiencing one to 4+ adversities before age 15 years.

**Table 2:** Hazard ratio's for bipolar risk after exposure to early-life events in the entire cohort and in cohort members with/without parental psychopathology

	*All*a,^b^			*No parental psychopathology*c,^b^			*Parental psychopathology*c,^b^		
	*HR*	*95% CI*		*HR*	*95% CI*		*HR*	*95% CI*	
*Exposures—age 0–14 years*									
Familial disruption^d^	2.02	1.84	2.22	1.80	1.61	2.00	4.07	3.59	4.60
Parental chronic somatic disorder^d^	1.71	1.52	1.92	1.46	1.27	1.69	3.77	3.19	4.46
Parental labour market affiliation^d^	2.55	2.23	2.91	1.97	1.63	2.39	4.25	3.61	5.01
Parental imprisonment^d^	2.09	1.77	2.47	1.94	1.54	2.45	3.01	2.41	3.76
Out-of-home care^d^	3.44	2.91	4.05	2.74	2.10	3.56	5.12	4.19	6.26
Parental loss to natural causes^d^	1.31	0.97	1.78	1.05	0.69	1.59	2.82	1.83	4.36
Parental loss to unnatural causes^d^	3.02	2.23	4.09	1.91	1.12	3.24	5.52	3.83	7.96

*Any exposure*									
Age 0–4 years	1.74	1.60	1.90	1.60	1.44	1.79	3.50	3.10	3.94
Age 5–9 years	1.55	1.41	1.70	1.48	1.31	1.68	3.21	2.78	3.69
Age 10–14 years	1.43	1.30	1.57	1.20	1.05	1.36	3.35	2.93	3.84
Age 0–14 years	1.90	1.74	2.07	1.68	1.52	1.86	3.92	3.50	4.39

*Number of exposures—age 0–14 years*									
0	1.00	Reference		1.00	Reference		3.12	2.60	3.75
1	1.68	1.53	1.85	1.59	1.43	1.78	3.63	3.15	4.19
2	2.30	2.03	2.60	1.91	1.62	2.24	4.30	3.65	5.07
3	2.77	2.31	3.32	2.14	1.60	2.85	4.34	3.47	5.42
4+	2.45	1.78	3.36	1.73	0.93	3.24	3.50	2.43	5.04

Abbreviations: CI, confidence interval; HR, hazard ratio.

a Hazard ratios were obtained by Cox regression and adjusted for calendar year and sex.

b Reference group for all results are those not exposed to any early-life event and not exposed to parental psychopathology.

c Hazard ratios were obtained by Cox regression on the interaction between early-life event and parental psychopathology status. adjusted for calendar year and sex.

d Individuals are considered exposed independent on whether this was a single exposure or an exposure combined with other life events.
