# Supplementary material for: Childhood adverse life events and parental psychopathology as risk factors for bipolar disorder
Source: Transl Psychiatry. 2016 Oct 25;6(10):e929–. doi: 10.1038/tp.2016.201 (PMC5290348; doi:10.1038/tp.2016.201)
Supplement: Supplementary Table 1 [file tp2016201x1.doc]

**Supplementary table 1: Hazard ratios for bipolar risk after exposure to early life events. stratified by parental psychopathology status.**

|  | **No parental psychopathology*** | | | **Parental psychopathology**** | | |
| --- | --- | --- | --- | --- | --- | --- |
|  | **HR** | **95% CI** | | **HR** | **95% CI** | |
| **Exposures - age 0-14 years** |  |  |  |  |  |  |
| Familial disruption | 1.80 | 1.61 | 2.00 | 1.30 | 1.08 | 1.57 |
| Parental chronic somatic disorder | 1.46 | 1.27 | 1.69 | 1.21 | 0.97 | 1.51 |
| Parental labour market affiliation | 1.97 | 1.63 | 2.39 | 1.36 | 1.09 | 1.69 |
| Parental imprisonment | 1.94 | 1.54 | 2.45 | 0.96 | 0.74 | 1.26 |
| Out-of-home care | 2.74 | 2.10 | 3.56 | 1.64 | 1.28 | 2.10 |
| Parental loss to natural causes | 1.05 | 0.69 | 1.59 | 0.90 | 0.57 | 1.43 |
| Parental loss to unnatural causes | 1.91 | 1.12 | 3.24 | 1.77 | 1.19 | 2.62 |

| * Hazard ratios were obtained by Cox regression on the interaction between early life event and parental psychopathology status. adjusted for calendar year and sex.  Reference group for all results are those not exposed to any early life event and not exposed to parental psychopathology. | |
| --- | --- |
| ** Hazard ratios were obtained by Cox regression on the interaction between early life event and parental psychopathology status. adjusted for calendar year and sex.  Reference group for all results are those not exposed to any early life event but exposed to parental psychopathology. |  |
